# Photoactivated adenylyl cyclase (PAC) reveals novel mechanisms underlying cAMP-dependent axonal morphogenesis

**DOI:** 10.1038/srep19679

**Published:** 2016-01-22

**Authors:** Zhiwen Zhou, Kenji F. Tanaka, Shigeru Matsunaga, Mineo Iseki, Masakatsu Watanabe, Norio Matsuki, Yuji Ikegaya, Ryuta Koyama

**Affiliations:** 1 Laboratory of Chemical Pharmacology, Graduate School of Pharmaceutical Sciences, The University of Tokyo, 7-3-1 Hongo, Bunkyo-ku, Tokyo, Japan; 2 Department of Neuropsychiatry, School of Medicine, Keio University, 35 Shinanomachi, Shinjuku, Tokyo, Japan; 3 Central Research Laboratory, Hamamatsu Photonics K.K., 5000 Hirakuchi Hamakita-ku, Hamamatsu, Shizuoka, Japan; 4 Faculty of Pharmaceutical Sciences, Toho University, 2-2-1 Miyama, Funabashi, Chiba, Japan; 5 The Graduate School for the Creation of New Photonics Industries, 1955-1 Kurematsu-cho, Nishiku, Hamamatsu, Shizuoka, Japan

## Abstract

Spatiotemporal regulation of axonal branching and elongation is essential in the development of refined neural circuits. cAMP is a key regulator of axonal growth; however, whether and how intracellular cAMP regulates axonal branching and elongation remain unclear, mainly because tools to spatiotemporally manipulate intracellular cAMP levels have been lacking. To overcome this issue, we utilized photoactivated adenylyl cyclase (PAC), which produces cAMP in response to blue-light exposure. In primary cultures of dentate granule cells transfected with PAC, short-term elevation of intracellular cAMP levels induced axonal branching but not elongation, whereas long-term cAMP elevation induced both axonal branching and elongation. The temporal dynamics of intracellular cAMP levels regulated axonal branching and elongation through the activation of protein kinase A (PKA) and exchange protein directly activated by cAMP (Epac), respectively. Thus, using PAC, our study for the first time reveals that temporal cAMP dynamics could regulate axonal branching and elongation via different signaling pathways.

Proper axonal morphogenesis is crucial to the establishment of functional neural circuits^1^. Axonal morphogenesis consists of two basic steps: branching and elongation. Initially, one of several short neurites polarizes and elongates to become a primary axon. Then, axonal branches are formed alongside the primary axon and elongate to form synapses with post-synaptic partners. The process of axonal morphogenesis is spatiotemporally regulated such that axons provide synaptic connections with proper targets at precise timing.

Among several intracellular signaling molecules, 3′-5′-cyclic adenosine monophosphate (cAMP) has been suggested to serve as a key mediator in axonal growth. For example, cAMP mediates axonal branching induced by neurotrophins such as brain-derived neurotrophic factor (BDNF) and NT3^2^. In addition, intracellular cAMP elevations induced by the activation of deleted in colorectal cancer (DCC), a receptor for the axon guidance cue Netrin-1, promoted axonal elongation *in vivo* and induced branch formation *in vitro*^3^,^4^. Despite extensive studies into the role of cAMP in axonal growth, little is known about whether and how changes in intracellular cAMP levels regulate axonal morphogenesis. One reason for this gap is that methods to spatiotemporally manipulate intracellular cAMP levels have been unavailable. Indeed, most studies have pharmacologically assessed the role of cAMP in axonal morphogenesis via the chronic application of cAMP agonists or antagonists to cultured neurons. These methods are insufficient to probe the effects of spatiotemporal cAMP dynamics on axonal morphogenesis.

To overcome these methodological issues, we utilized an optogenetic method. Specifically, we used photoactivated adenylyl cyclase (PAC), which was originally identified as a sensor for photoavoidance in the flagellate *Euglena gracilis*^5^. PAC, which consists of two PACα and two PACβ subunits, is activated by blue light and rapidly changes its conformation to synthesize cAMP from adenosine triphosphate (ATP). Previous studies have shown that PAC produces cAMP in response to blue light and increases intracellular cAMP levels when expressed in *Aplysia* sensory neurons^6^, *Xenopus* oocytes, mammalian cells, and adult fruit flies^7^. Further, an *in vitro* study reported that the activation of PAC in euglena cells enabled the precise temporal manipulation of intracellular cAMP levels^8^.

To investigate whether and how intracellular cAMP dynamics regulates axonal elongation and branching, we transfected PAC in primary cultures of the dentate granule cells. We successfully performed temporal manipulation of intracellular cAMP levels in PAC-transfected granule cells by changing the time-course of blue-light exposure. We found that short-term elevation of intracellular cAMP induces axonal branching but not elongation, whereas long-term cAMP elevation induces both axonal branching and elongation. Furthermore, using PAC together with pharmacological tools and siRNA-mediated knockdown, we found that intracellular cAMP regulates axonal branching and elongation via protein kinase A (PKA) and exchange protein directly activated by cAMP (Epac), respectively.

## Results

### PAC activation elevated intracellular cAMP levels in cultured neurons

**Figure 1:**
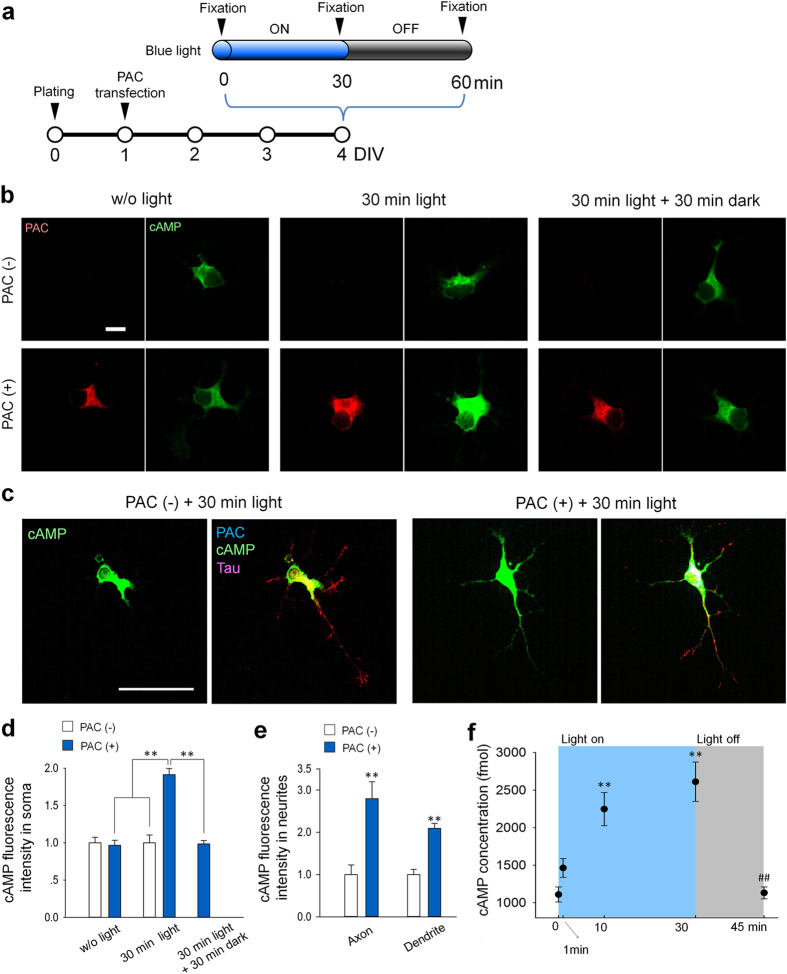
PAC activation elevates intracellular cAMP levels. (**a**) Experimental paradigms for cAMP quantification via immunocytochemistry. (**b**) Representative images of cAMP fluorescence intensity in cultured granule cells immunostained for PAC (red) and cAMP (green). (**c**) Representative image of granule cells immunostained for the microtubule associated protein tau (red), cAMP (green), and PAC (blue) in 30 min light group. The images of a PAC negative cell (PAC (−)) are shown on the left, while the images of PAC positive cell (PAC (+)) are shown on the right. Scale bars = 10 μm. (**d**) Quantification of somatic cAMP fluorescence intensity in cultured granule cells. The cAMP fluorescence intensity was normalized to that of PAC (−) granule cells in each group. cAMP levels returned to basal levels 30 min after the light was turned off (30 min light +30 min dark). **p < 0.01 between indicated groups; Tukey’s test after one-way ANOVA, n = 27–68 cells. (**e**) Quantification of cAMP fluorescence intensity in the axonal and dendritic neurites of cultured granule cells 30 min after the light stimulation. The cAMP fluorescence intensity was normalized to that of PAC (−) granule cells in each group. **p < 0.01 vs. PAC (−) cells; Tukey’s test after one-way ANOVA , n = 11–19 cells. (**f**) Intracellular cAMP levels were analyzed using an ELISA of PAC-transfected HEK cell lysates at each time point after blue-light stimulation. **p < 0.01 vs. 0 min, and ^##^*p* < 0.01 vs. 30 min; Tukey’s test after a one-way ANOVA, n = 4–8 wells.

First, we examined the properties of PAC (photoactivated adenylyl cyclase) in cultured dentate granule cells (Fig. 1a). Primary cultures of the dentate granule cells were prepared from P3-4 rats^9^ and transfected with mCherry-2A-PACα at day 1 *in vitro* (DIV 1). At DIV 4, the cultured neurons were stimulated with pulsed blue light (2 s on/3 s off, maximum wavelength of 470 nm) for various durations and subsequently immunostained for PAC and cAMP. cAMP fluorescence intensity in Fig. 1d was normalized to the fluorescence of PAC (−) cells. Blue-light illumination did not affect the cAMP fluorescence intensity in neurons lacking PAC expression (Fig. 1b,d, the cAMP fluorescence of PAC (−) +30 min light (n = 31) = 0.9 ± 0.11, normalized to PAC (−) w/o light (n = 27), no significant difference). We also confirmed that PAC expression alone did not affect cAMP fluorescence intensity without light illumination (Fig. 1b,d, w/o light), though PAC has been reported to possibly exhibit cyclase activity in the dark^7^. In PAC-expressing neurons, PAC was mainly localized in the soma, and 30 min of blue-light illumination significantly increased cAMP fluorescence intensity in the soma (Fig. 1b–d, 30 min light). The cAMP immunofluorescence was also detected in axonal and dendritic neurites (Fig. 1c, PAC (+) +30 min light, and Fig 1e). The increased cAMP signals returned to basal levels when neurons were fixed 30 min after the 30-min light illumination (Fig. 1b,d, 30 min light +30 min dark).

We also quantified PAC-induced cAMP dynamics by performing ELISA on the lysates from PAC-transfected HEK293T cells. Following a 1-min light illumination, cAMP levels increased slightly compared with the no-light-exposure group (0 min) (Fig. 1f). Following 10 or 30 min of light exposure, however, cAMP levels increased significantly compared with the no-light-exposure group (0 min). The elevated cAMP levels decreased to basal levels 15 minutes after the light was turned off (Fig. 1f). These results indicate that blue-light illumination rapidly activates PAC to synthesize cAMP and that PAC rapidly returns to its inactivated state in the dark.

### Transient PAC activation induced axonal branching and elongation

**Figure 2:**
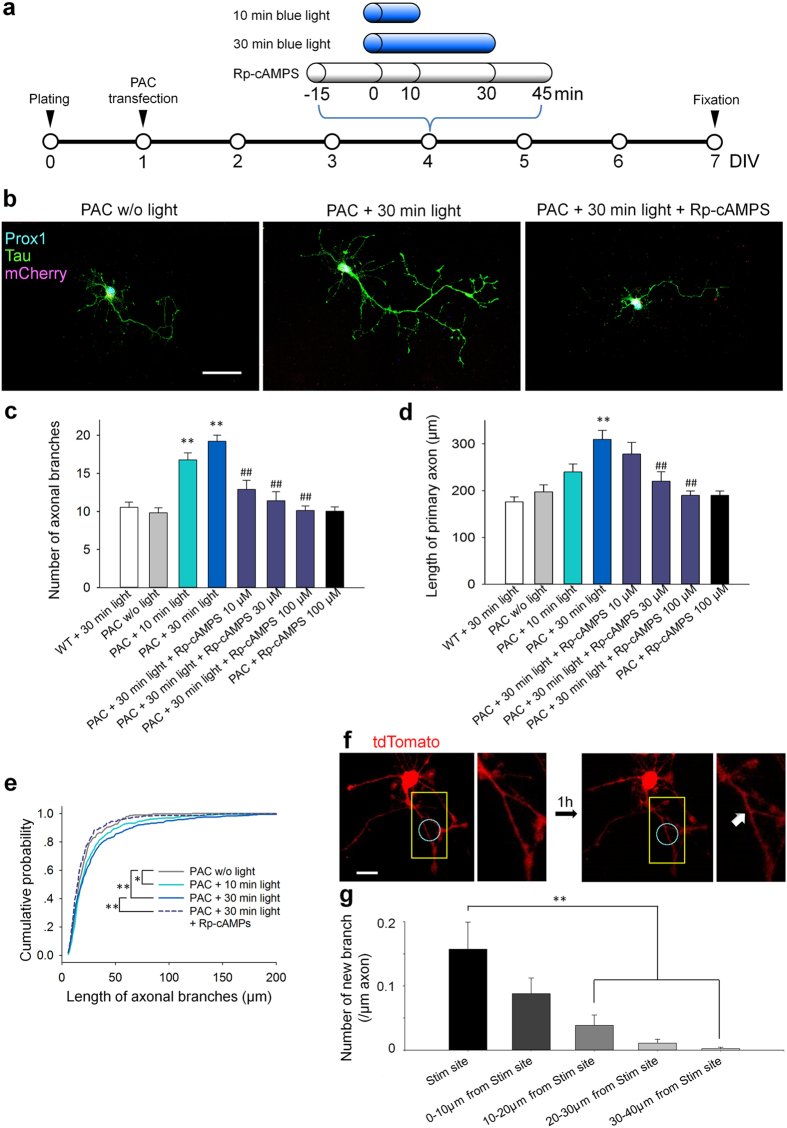
Transient PAC activation induces axonal branching and elongation. (**a**) Experimental paradigms for the axonal morphology assay. Rp-cAMPS was bath-applied at 100 μM for 1 hr 15 min before the light exposure. (**b**) Representative images of cultured granule cells immunostained for mCherry (red), tau (green) and Prox1 (blue). Scale bar = 50 μm. (**c**) Bar graphs indicating the number of branches. **p < 0.01 vs. PAC w/o light, and ^##^*p* < 0.01 vs. PAC + 30 min light; Steel-Dwass test after Kruskal-Wallis test, n = 30 cells for each group. (**d**) Bar graphs indicating the length of the primary axon. **p < 0.01 vs. PAC w/o light, and ^##^*p* < 0.01 vs. PAC + 30 min light; Tukey’s test after a one-way ANOVA, n = 30 cells for each group. (**e**) Cumulative probability of the length of each axonal branch. *p < 0.05 and **p < 0.01; Kolmogorov–Smirnov test, n = 200–600 branches from 30 cells for each group. (**f**) Representative images of a PAC- and tdTomato-expressing granule cell before (left) and 1 h after (right) focal light stimulation. The cyan circle indicates the stimulated region. Magnified images of areas inside the yellow boxes are shown below. The white arrow indicates a newly formed branch. Scale bars = 20 μm. (**g**) A bar graph showing the probability of new branch formation around the stimulated region is shown. The probability was higher near or inside the stimulation site. **p < 0.01 between indicated groups, Tukey’s test after a one-way ANOVA, n = 55 trials from 10 cells.

Next, we stimulated PAC-transfected granule cells with either 10 or 30 min of blue-light illumination at DIV 4 and examined morphological changes in the neurons 72 hours later (DIV 7) (Fig. 2a). We confirmed that light alone did not change axonal morphology (the total length, branch number and length of the primary axon were not changed, Fig. 2c,d, white bars). PAC expression in the granule cell was confirmed by co-labeling with mCherry and Prox1, a granule cell marker. The neurites were visualized using tau-1 immunofluorescence (Fig. 2b; see also Supplementary Fig. 2a for split channels). The longest neurite that emerged from the soma was defined as the primary axon, and collaterals emerging from the primary axon were defined as collateral branches. First, we examined the effect of PAC activation on the number of axonal branches. We found that both 10 and 30 min of blue-light illumination significantly increased the number of axonal branches compared with no light (Fig. 2b,c). Second, we examined the effect of PAC activation on the primary axon length. We found that 30 min of light illumination significantly increased the axonal length compared with the control group, whereas 10 min of light illumination had no significant effect (Fig. 2d). Importantly, these phenomena were blocked by bath application of the cAMP antagonist Rp-cAMPS (100 μM), indicating that PAC activation-induced axonal morphogenesis was mediated by intracellular cAMP (Fig. 2b–d). Additionally, we confirmed that Rp-cAMPS alone affected neither axonal branching nor elongation in our culture conditions. We also found that lower concentration of Rp-cAMPS (10 μM and 30 μM) blocked axonal branching, while 10 μM of Rp-cAMPS did not block cAMP-induced axonal elongation (Fig. 2c,d). Finally, the influence of cAMP on axonal elongation was also confirmed by a light- and time-dependent increase in the lengths of the axonal branches (Fig. 2e).

Another advantage of the use of PAC is the manipulation of local cAMP levels in subcellular compartments via application of focal light stimulation. We therefore applied light stimulation to a restricted axonal region in PAC-transfected granule cells and examined axonal morphology 1 h after stimulation. On DIV 1, neurons were cotransfected with PAC and membrane-targeted tdTomato, which enabled the clear visualization of cell shape, and light stimulation was performed between DIVs 4 and 6. In 14 of 55 trials, new branches formed within the stimulated region (Fig. 2f, cyan circles). We quantified the probability of branch formation in the axonal regions and found that axons tended to exhibit branching near the stimulated region (Fig. 2g).

The effect of cAMP on neuronal morphology was also validated using classical pharmacological experiments (Supplementary Fig. 1). Either the cAMP agonist Sp-cAMPS or the cAMP antagonist Rp-cAMPS was bath-applied to the culture medium, and the resulting axonal and dendritic morphologies were analyzed immunocytochemically (Supplementary Fig. 1a,b). Sp-cAMPS (100 μM) significantly increased the number of axonal branches but not the length of the primary axon (Supplementary Fig. 1c,d), which suggests that the bath application of cAMP analogues acts differently than intracellular cAMP because the PAC-induced increase in intracellular cAMP resulted in the elongation of primary axons as well (Fig. 2d). Rp-cAMPS (100 μM) affected neither the branch number nor the axonal length (Supplementary Fig. 1b–d). Additionally, Sholl analysis of the dendritic complexity of the cultured granule cells revealed that neither Sp-cAMPS nor Rp-cAMPS affected dendritic morphology (Supplementary Fig. 1e), which is not surprising because the role of cAMP in dendritic morphogenesis has been reported to be less prominent compared with cyclic GMP^10^.

### Roles of PKA and Epac in axonal morphogenesis

**Figure 3:**
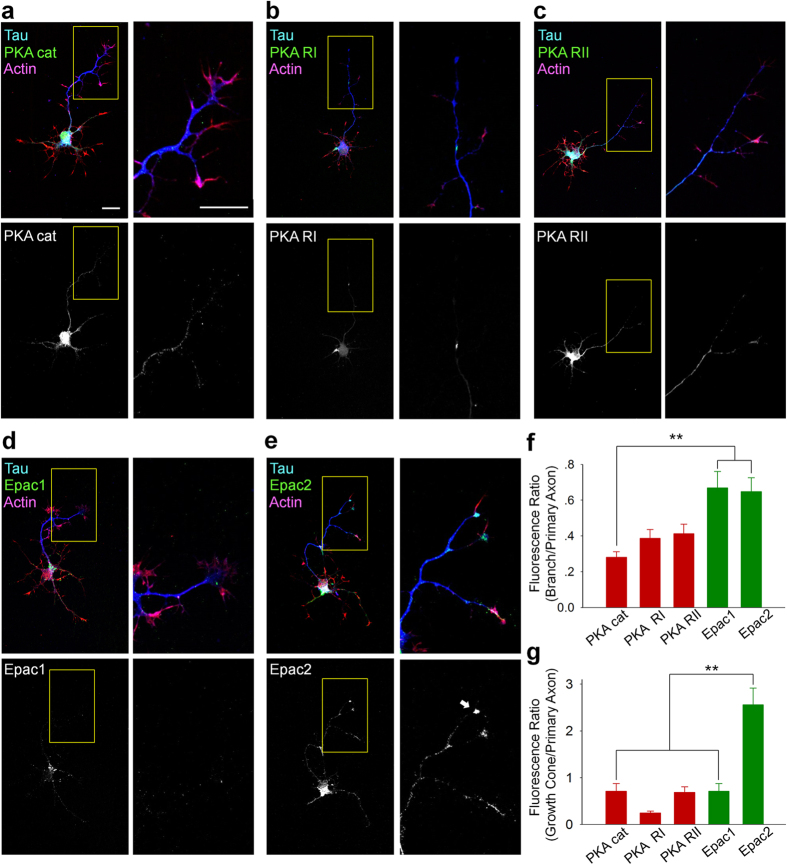
Subcellular expression of PKA and Epac in the axonal region. (**a**–**e**) Left, Representative images of cultured granule cells at DIV 4, immunolabeled by antibodies against PKA catalytic α/β subunits (**a**, PKA cat), PKA regulatory domain I (**b**, PKA RI), PKA regulatory domain II α (**c**, PKA RII), Epac1 (**d**), or Epac2 (**e**). Axon was immunolabeled by anti-Tau antibody and a whole cell morphology (actin) was labelled by rhodamine phalloidin. Right, Magnified images of the boxed region in the left panels. Scale bar = 20 μm. (**f**) Quantifications of the subcellular distribution of PKA cat, PKA RI, PKA RII, Epac1, and Epac2 in branches. The ratio of fluorescence intensity in the axonal branches to that of the primary axonal shafts reveals that PKA localized more in primary axons than in axonal branches, whereas both Epac1 and Epac2 exhibited higher localization in branches. **p < 0.01 between indicated groups; Tukey’s test after one-way ANOVA, n = 10 cells for each group. (**f**) Quantifications of the subcellular distribution of PKA cat, PKA RI, PKA RII, Epac1, and Epac2 in growth cones. The ratio of the growth cone fluorescence intensity to that of the primary axonal shafts demonstrates the strong accumulation of Epac2 in growth cones. **p < 0.01 between indicated groups; Tukey’s test after one-way ANOVA, n = 10 cells for each group.

Our results indicated that an elevated intracellular cAMP levels are sufficient to trigger both axonal branching and elongation. cAMP mediates various signaling cascades through two major targets: protein kinase A (PKA) and exchange protein directly activated by cAMP (Epac). Thus, we next examined whether and how PKA and Epac are involved in cAMP-induced axonal branching and elongation. First, we immunocytochemically determined the axonal localization of PKA (PKA cat, the catalytic subunit of PKA; PKA RI, the regulatory subunit I of PKA; PKA RII, the regulatory subunit II of PKA) and Epac (Epac1 and Epac2) in cultured granule cells (Fig. 3). Cultured granule cells were fixed on DIV 4, which was in consistent with the time when light stimulation was applied in other experiments. Cellular morphology was visualized by co-labeling of tau and filamentous actin (F-actin). We found that axonal PKA (both catalytic and regulatory subunits) expression was mainly confined to axonal shafts (Fig. 3b,e,f). We did not find a strong localization of PKA cat or PKA RI in the growth cone, whereas PKA RII was found in the growth cone but the expression level was low compared to axonal shaft. In contrast to PKA, both Epac1 and Epac2 expression in branches were comparable to their expression at axonal shaft (Fig. 3e). Notably, Epac2 was also highly localized in growth cones, and was detected in minor neurites (Fig. 3e–g).

**Figure 4:**
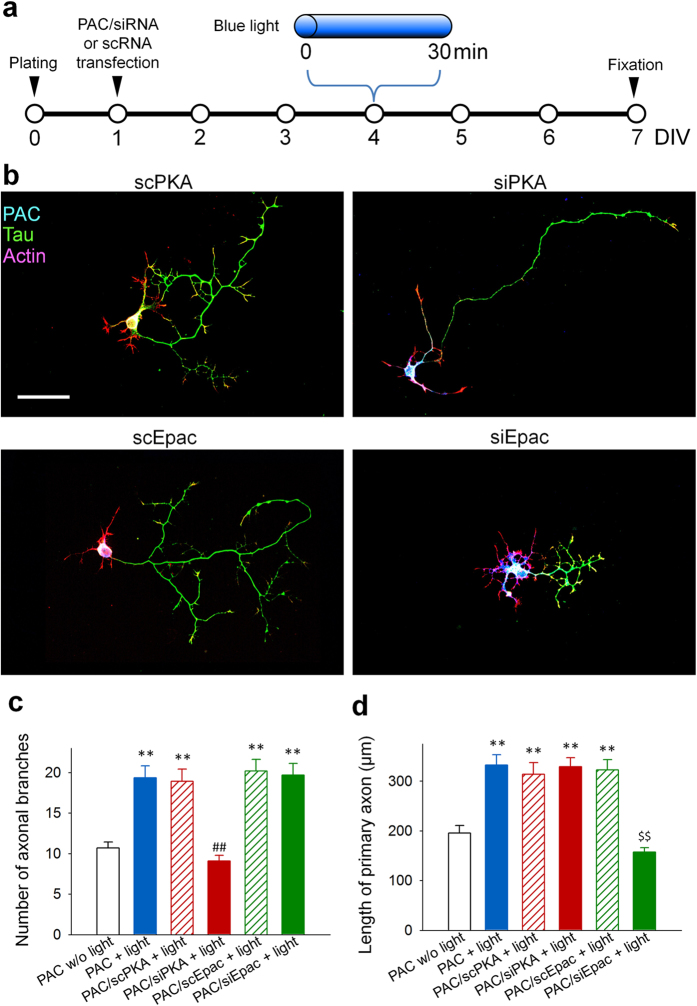
PKA and Epac knockdown abolished cAMP-induced axonal branching and elongation, respectively. (**a**) Experimental paradigms for the axonal morphology assay. (**b**) Representative images of cultured granule cells immunostained for tau (green) and PAC (blue). Actin was labeled with rhodamine phalloidin (red). Scale bar = 50 μm. (**c**) Bar graphs indicating the number of branches. **p < 0.01 vs. PAC w/o light, and ^##^*p* < 0.01 vs. PAC/scPKA + light; Steel-Dwass test after Kruskal-Wallis test, n = 20–30 cells for each group. (**d**) Bar graphs indicating the length of the primary axon. **p < 0.01 vs. PAC w/o light, and ^##^*p* < 0.01 vs. PAC/scPKA + light; Tukey’s test after a one-way ANOVA, n = 20–30 cells for each group.

Next, we directly examined the involvement of PKA and Epac in axonal morphogenesis using the small interference RNA (siRNA)-mediated knockdown of PKA and Epac in PAC-transfected granule cells (Fig. 4). A cocktail of siRNAs against both PKA α and β subunits (siPKA) was used to knockdown PKA, and a cocktail of siRNAs against Epac1 and Epac2 (siEpac) was used to knockdown Epac. Scrambled control RNAs (scRNAs) were designed based on the sequences of the PKA subunits (scPKA) or the Epacs (scEpac) and used for control experiments. The knockdown efficiency of the siRNAs was immunocytochemically validated (Supplementary Fig. 2). When cultured granule cells were co-transfected with PAC and either scPKA or scEpac, 30 min of blue-light illumination at DIV 4 induced a significant increase in the number of axonal branches and the length of primary axon that was comparable to PAC-expressing cells with blue-light illumination (PAC + light) (Fig. 4b–d; see also Supplementary Fig. 2b for split channels of Fig. 4b images). Notably, the granule cells transfected with siPKA exhibited significantly longer primary axons without excessive branching in response to blue-light illumination (Fig. 4b–d). In contrast to the cells transfected with siPKA, the cells transfected with siEpac exhibited significantly higher numbers of axonal branches without distinct elongation of the primary axons (Fig. 4b–d). Together with the localization of PKA in axonal shafts and Epac in axonal branches and growth cones (Fig. 3), these results suggest that PKA activation in the axonal shaft is required for axonal branching, probably via promoting local accumulation of actin filaments and transportation of microtubules into filopodia, both of which take place in the axonal shaft and required for membrane protrusion, i.e., the initial step for branch formation^1^, whereas the activation of Epac, which highly localized in the growth cone, a leading tip of an axon, is required for axonal elongation.

We also pharmacologically validated the roles of PKA and Epac using 6-phe-cAMP (a PKA activator, 200 μM) and 8-cpt-2Me-cAMP (an Epac activator, 200 μM), respectively (Supplementary Fig. 3). We found that chronic pharmacological treatment did not fully reproduce the changes in axonal morphogenesis induced by the temporally regulated intracellular cAMP levels (Fig. 4).

### Temporal cAMP dynamics regulates axonal morphogenesis

**Figure 5:**
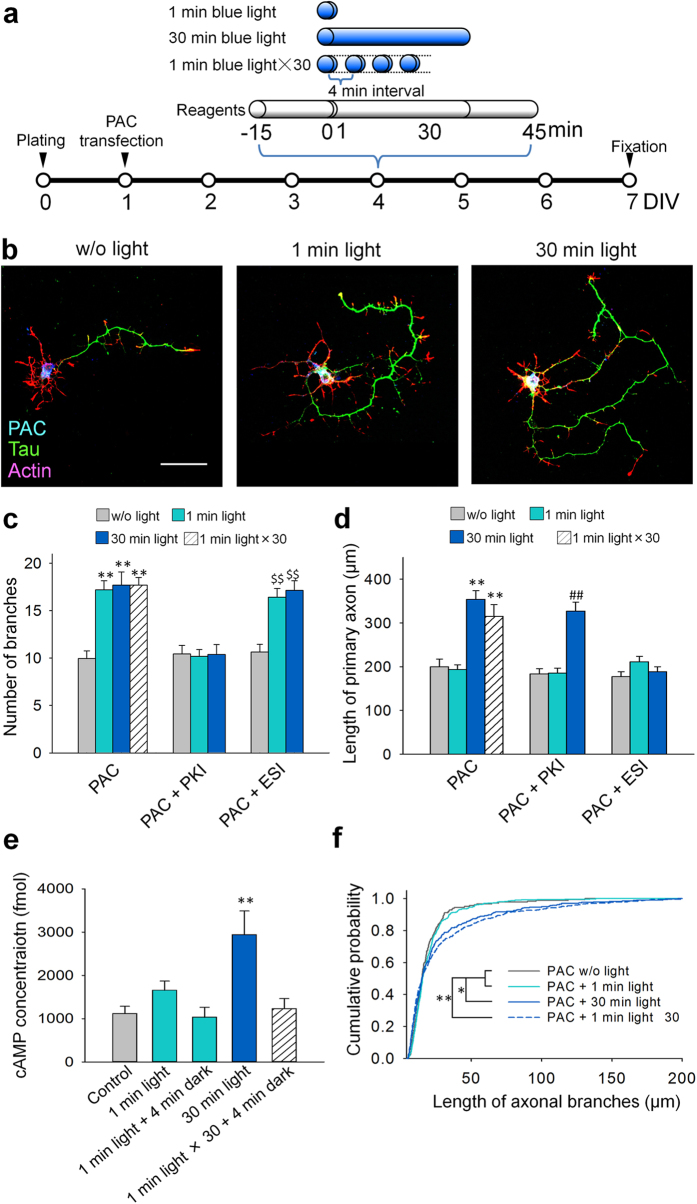
Temporal cAMP dynamics regulates axonal morphogenesis. (**a**) Experimental paradigms. In some cases, blue light was applied with a protocol of 1 min × 30 times with 4-min intervals. For reagents, either the PKA antagonizing peptide PKI (20 μM) or the Epac blocker ESI-09 (100 μM) was bath-applied 15 min before light exposure for 1 hr. (**b**) Representative images of cultured granule cells immunostained for tau (green) and PAC (blue). Actin was labeled with rhodamine phalloidin (red). Scale bar = 50 μm. (**c**) Bar graphs indicating the number of branches. **p < 0.01 vs. PAC w/o light, ^##^*p* < 0.01 vs. PAC PKI w/o light, and ^$$^*p* < 0.01 vs. PAC ESI w/o light; Steel-Dwass test after Kruskal-Wallis test, n = 20–30 cells for each group. (**d**) Bar graphs indicating the length of the primary axon. **p < 0.01 vs. PAC w/o light, ^##^*p* < 0.01 vs. PAC PKI w/o light, and ^$$^*p* < 0.01 vs. PAC ESI w/o light; Tukey’s test after a one-way ANOVA, n = 20–30 cells for each group. (**e**) Intracellular cAMP levels were analyzed with an ELISA of PAC-transfected HEK cell lysates. **p < 0.01 vs. control; Tukey’s test after a one-way ANOVA, n = 4–8 wells. (**f**) Cumulative probability of the length of each axonal branch. *p < 0.05 and **p < 0.01; Kolmogorov–Smirnov test, n = 150–500 branches from 20–30 cells for each group.

To further determine the effects of temporal cAMP dynamics, we first exposed PAC-expressing granule cells to either 1 or 30 min of blue light (Fig. 5a). We found that 1 min of light illumination increased the number of axonal branches without affecting the axonal length, whereas 30 min of light illumination significantly increased both axonal length and the number of branches (Fig. 5b–d). These results raised the possibility that 1 min of blue-light illumination activated only PKA to induce axonal branching and that 30 min of light illumination activated both PKA and Epac to induce axonal branching and elongation.

We hypothesized that the activation of PKA and Epac is regulated by differences in intracellular cAMP concentrations and/or the length of time during which the intracellular cAMP levels are elevated. Indeed, previous studies have shown that the cAMP-binding affinity of Epac is at least tenfold lower than that of PKA^11^ and that PKA activity saturated at low cAMP concentrations while Epac activity did not saturate even at twofold higher cAMP levels^12^. To test this hypothesis, we first performed an ELISA using HEK293T cells (Fig. 5e) and found that 30 min of light illumination significantly increased intracellular cAMP levels. By contrast, the effect of 1 min of light illumination was moderate, even though a slight increase in cAMP levels was detected (Fig. 5e). Second, we pharmacologically inhibited PKA or Epac (Fig. 5c,d) with a synthetic peptide inhibitor of PKA (PKI) or the recently developed selective Epac blocker ESI-09^13^, respectively. PKA inhibition with PKI blocked axonal branching induced by both 1 min and 30 min of light exposure (Fig. 5c), but PKI failed to block 30 min of light-induced axonal elongation (Fig. 5d). By contrast, Epac inhibition with ESI-09 did not block axonal branching induced either by 1 min or 30 min of light exposure (Fig. 5c). However, ESI-09 blocked 30 min of light-induced axonal elongation (Fig. 5d).

These results suggest that lower and shorter elevations in intracellular cAMP concentrations, i.e., 1 min of light exposure, induce axonal branching via PKA and that higher and longer elevations of intracellular cAMP concentration, i.e., 30 min of light exposure, induce axonal elongation via Epac. However, it remained unclear whether high elevations of intracellular cAMP concentrations, sustained elevations of cAMP, or both, are necessary to induce axonal elongation. To answer this question, we performed another time-course of blue-light illumination: the cultured cells were exposed to 1 min of blue light 30 times, with 4-min intervals between each exposure (Fig. 5a). An ELISA confirmed that 1 min of light exposure moderately increased cAMP concentrations (not statistically significant) and that cAMP levels returned to the baseline 4 min later (Fig. 5e). In addition, cAMP concentrations also returned to baseline 4 min after the last (30^th^) exposure of blue light (Fig. 5e). Thus, the application of 30 1-min light exposures induced a prolonged and modest elevation of intracellular cAMP levels. The 1-min light × 30 protocol induced both axonal branching and elongation that were comparable to the morphological changes induced by 30 min of continuous light exposure (Fig. 5c,d). Finally, the cumulative probability of the length of each axonal branch confirmed that both the 30-min and the 30 × 1-min protocols resulted in the elongation of axonal branches, whereas 1 min of exposure did not affect the length (Fig. 5f).

We have further performed videomicroscopy experiments (images were taken every 5 min for 30 min after the light stimulation, using 1 min and the 30 min paradigms) to examine if a brief cAMP increase might enhance axon elongation during a short period of time in Fig. S5. We did not find any sign of enhanced axonal elongation during the monitored time course (Fig. S5b). Further, we have immunocytochemically assessed axonal morphology 1 hr after the light stimulation, finding no significant changes in axonal branch number (Fig. S5c) and axonal length (Fig. S5d) compared to control (no light) group. These findings rule out the short term effect of a brief elevation of cAMP on axon elongation.

Together, these results suggest that a sustained elevation of intracellular cAMP levels is necessary to induce axonal elongation.

The use of PAC allowed us to manipulate intracellular cAMP levels, and, taken together, our findings revealed novel mechanisms by which temporal cAMP dynamics regulate branching and elongation via PKA and Epac, respectively.

## Discussion

Accumulating evidence has shown that cAMP plays an important role in axonal morphogenesis and neural circuit formation. However, the influence of temporal changes in intracellular cAMP levels on axonal morphogenesis has remained unclear, and little is known about whether and how cAMP regulates axonal branching and elongation separately. In the present study, we used photoactivated adenylyl cyclase (PAC) to determine that the temporal modulation of intracellular cAMP is crucial in axonal branching and elongation via PKA and Epac, respectively.

Since its discovery, PAC has been used to widen our understanding of the role of cAMP in physiological conditions. Compared with cAMP analogues such as Sp-cAMPS, the use of PAC is advantageous, particularly because PAC enables the temporal regulation of intracellular cAMP levels. Changes in intracellular cAMP levels are often dynamic. For example, previous work has shown that intracellular cAMP levels are periodically regulated according to circadian rhythms in cultured suprachiasmatic nuclei, a brain region assumed to be the “circadian clock”^14^. Another study has shown that cAMP oscillations with transient, but not constant, elevation induced synapse elimination in developing retinal ganglion cells^15^. Thus, temporal regulation of intracellular cAMP levels, which cannot be achieved by chronic application of pharmacological reagents, is necessary to study the physiological role of cAMP.

Spatial regulation of intracellular cAMP was also achieved through the use of PAC (Fig. 2f,g). Spatially restricted cAMP transients have been suggested to be particularly important in neuronal connectivity^16^. The spatial localization of PKA and Epac was different in the cultured granule cells: PKA localized to the axonal shaft, whereas Epac localized to the growth cones (Fig. 3). Therefore, it is possible that cAMP elevations in specific subcellular regions regulate axonal morphogenesis via the sole activation of either PKA or Epac. In future, the molecular tagging and expression of PAC in subcellularly localized regions such as growth cones would help address these questions. In addition, the local activation of PAC is of great interest for elucidating the influence of focal cAMP transients on axonal morphogenesis.

Though we demonstrated that the subcellular activation of PAC in axons also induces axonal branching (Fig. 2f,g), we do not claim that axonal cAMP alone is responsible for axonal elongation and branching in our experimental system, in which blue-light illumination was applied to whole cell but not subcellularly. Thus, we do not exclude the possible involvement of cAMP signalling in soma or minor dendritic neurites in axonal morphogenesis. Indeed, axonal branching and elongation were not drastically induced 1h after light stimulation (Fig. S5). These results imply that cAMP-induced axonal branching and elongation may require gene transcription and protein synthesis, the former mainly taking place at soma. For example, the activation of cAMP-responsive element binding protein (CREB), a transcription factor activated by cAMP, has been shown to be crucial for axon growth^17^. On the other hand, to our knowledge, there is no evidence so far that indicates an elevation of cAMP in minor dendrites alone could affect axonal branching or elongation. In addition, in the present study, a 72 h application of Sp-cAMPS did not influence the dendritic complexity (Fig. S1e). It would be interesting to examine the role of dendritic cAMP in axonal morphogenesis in the future.

In this study, we examined whether PKA and Epac, major downstream targets of cAMP, differentially regulate axonal morphogenesis. We found that PKA activation triggers axonal branching and that Epac activation induces axonal elongation. PKA is a well-studied downstream target of cAMP and is thought to mediate the polymerization and elongation of actin filaments in response to axon guidance cues such as Netrin-1 and neurotrophins^2^,^18^. Thus, PKA activation-induced axonal branching in the present study is likely mediated by the increasing protrusive activity of filopodia, which is crucial in branch formation^19^. This model is consistent with our findings on the subcellular expression of PKA (Fig. 3) because protrusive filopodia are formed alongside primary axons^20^. Epac, by contrast, was discovered as a GTP exchange factor for the small G protein Rap1^21^,^22^, and its role in axonal morphogenesis has been studied^23^,^24^. The activation of Epac has been shown to induce neurite outgrowth in PC12 cells^25^ and to enhance neurite regeneration in the adult rat spinal cord^24^.

Even though Epac has many downstream targets and most of them are signaled through Rap1 and Rap2, the Epac-dependent activation of the mitogen-activated family member c-jun N-terminal kinase (JNK)^26^, the small GTPase Rit^27^, and microtubule growth^28^ can be independent of Rap activation. Importantly, all of these processes are considered crucial for axon growth. For example, the phosphorylation of JNK has been indicated to be essential in axonal elongation^29^ and the expression of constitutively activated Rit mutant promoted axon growth^30^. The Epac-induced axonal elongation observed in the present study may also occur via microtubules, given that Epac activation increases microtubule length in human endothelial cells^28^ and interacts with microtubule-associated protein 1B^31^, which plays an important role in microtubule dynamics in axonal growth cones^32^.

It has been suggested that both PKA and Epac agonists induce multiple axons in cultured neurons^10^,^23^,^33^. However, pharmacological activation of PKA and Epac did not induce multiple axons in the present study. The difference between our and previous findings might results from the differences in the experimental system such as the type of neurons used, culture conditions, and the stimulus condition to activate PKA and Epac. For example, we used the dentate granule cells from P3-4 rats, whereas Shelly *et al*. used E18 rat hippocampal neurons^10^,^33^ and Muñoz-Llancao *et al*. used both hippocampal and cortical neurons prepared from E18 rats and mice^23^.

In the present study, we used Rp-cAMPS as a blocker of all cAMP pathways. Rp-cAMPS is recognized as a potent PKA antagonist, but its effect as a blocker of Epac is rather poor. Thus, we used Rp-cAMPS at 100 μM to inhibit cAMP signaling, because it has been reported that 100 μM Rp-cAMPS block approximately 50% of Epac activity^34^. We speculate that 50% inhibition of Epac activity was sufficient to block axon elongation, considering that long-term elevation of intracellular cAMP was necessary to trigger axon elongation through Epac pathway (Fig. 5d). This hypothesis was tested by experiments examining how a reduced concentration of Rp-cAMPS (10 μM and 30 μM) modulates PAC-induced axonal elongation and branching (Fig. 2c,d). We found that 10 μM Rp-cAMPS rescued axon elongation (Epac-dependent pathway) but still blocked the addition of axonal branching (PKA-dependent pathway). These results supports our hypothesis and further demonstrated that that the partial inhibition of Epac is sufficient to prevent axon elongation.

Our data indicated that cAMP plays important roles in axonal morphogenesis. Therefore, it was interesting that Rp-cAMPS alone did not affect neuronal morphogenesis (Fig. S1). We presume that Rp-cAMPS did not affect axonal morphology because the intracellular cAMP levels are basally low in cultured granule cells. Indeed, it has been reported that neuronal cAMP levels dramatically decrease after birth^35^. *In vivo*, intracellular cAMP levels would rapidly increase to initiate axonal branching and elongation, responding to the extracellular stimuli such as axon guidance cues and neurotransmitters. *In vivo*, neurons receive various extracellular signals that govern intracellular cAMP dynamics and result in axon morphogenesis towards network formation. In *X. laevis* embryos, blue-light stimulation of the PAC-expressing growth cones of spinal commissural axons modulated Netrin-1-dependent pathfinding^36^. The use of PAC as a tool to regulate cAMP levels *in vivo* will help reveal the mechanisms of network formation and the role of cAMP in developing neurons.

## Methods

### Primary culture of dentate granule cells

All experiments were performed with the approval of the animal experiment ethics committee at the University of Tokyo and according to the University of Tokyo’s guidelines for the care and use of laboratory animals. Cultures of dissociated granule cells were prepared from postnatal three-to-four-day-old (P3-4) SD rats as previously described^9^,^37^. Briefly, the dentate gyri were dissociated in ice-cold Gey’s balanced salt solution (GBSS) enriched with D-glucose (6.50 g/L) and subsequently treated with 0.25% trypsin and 0.01% DNase I at 37 °C for 30 min. After trypsinization was stopped via the addition of horse serum, cells were centrifuged at 1200 rpm for 5 min. The supernatants were removed, and the remaining cells were dispersed in 2 ml of culture medium with arabinofuranosyl cytidine and serum at 37 °C. For primary cultures, the dissociated granule cells were plated onto 13-mm cover slips coated with poly-D-lysine at a cell density of 5.0 × 10^3^ cells/cm^2^ in culture medium in 24-well plates and incubated at 37 °C in a humidified 5% CO_2_ and 95% air atmosphere. Medium was changed at DIV 1 and every 3 days after. The culture plates were wrapped with black tape to avoid exposing the PAC-transfected cells to light. For real-time imaging, cells were plated on glass-bottomed 35 mm dishes instead of 24-well plates.

### HEK cell culture

HEK293T cells were cultured in 500 μl Dulbecco’s Modified Eagle’s Medium (DMEM) with 10% fetal bovine serum in 24-well plates. Cells were plated at a density of 7.5 × 10^4^ cells/cm^2^.

### DNA and siRNA transfection

For granule cells, DNA constructs and siRNAs were transfected using Lipofectamine 2000 according to the manufacturer’s instructions. For one well of a 24-well plate, 0.4 μg of DNA or 20 pmol of siRNA was first dissolved in 50 μl OptiMEM I Reduced-Serum Medium as solution A, and 0.75 μl Lipofectamine 2000 was added to 50 μl OptiMEM as solution B. Solutions A and B were mixed, added to 400 μl of transfection culture medium (95% Neurobasal medium, 2 mM L-glutamine, and 1 mM sodium pyruvate at 37 °C in a humidified 5% CO_2_ and 95% air atmosphere) and used to replace the culture medium. The mixed solution was replaced with culture medium after 1 h.

For HEK cells, DNA constructs were transfected using Lipofectamine 2000 when the cells reached 80–100% confluency. For one well of a 24-well plate, 1.6 μg of DNA was dissolved in 100 μl OptiMEM I Reduced-Serum Medium as solution A, and 3.2 μl of Lipofectamine 2000 was dissolved in 100 μl OptiMEM as solution B. Solutions A and B were mixed after incubating for 5 min at room temperature. The mixed solution was added to 300 μl DMEM after a 20-min incubation, and the culture medium was subsequently replaced with this mixture. The transfection solution was replaced with DMEM supplemented with 10% FBS after 48 h.

DNA constructs for PACα, mGFP and membrane-tdTomato, as well as siRNAs against PKAα, PKAβ, EPAC1, and EPAC2 (plus scrambled control for each) were used. The membrane-GFP and membrane-tdTomato constructs were from Clontech. mCherry-2A-PACα was expressed under the control of the EF1α promoter. Plasmid information is available upon request.

### siRNAs

siRNAs were designed using the Sigma Genosys siRNA Service. The siRNA targeting the coding region of mouse PKAα (siPKAα) had the following sequence: sense, 5′rAUrGrCrAUrCUrArAUUUrArACrAUrCrCrATT; antisense, 5′UrGrGrAUrGUUrArArAUUrArGrAUrGrCrAUTT. The scrambled control siRNA for PKAα (scPKAα) was designed with the following sequence: sense, 5′rArArCrArCrAUUUrArAUrCrGrAUUUrArCrCTT; antisense, 5′rGrGUrArArAUrCrGrAUUrArArAUrGUrGUUTT. siPKAβ had the following sequence: sense, 5′UrGrGUrArGrCrGUUrGUrAUrAUUrATT; antisense, 5′UrArAUrAUrArCrArArCrGrCUrArCrCrATT. scPKAβ was designed with the following sequence: sense, 5′UUUrArArGUUrCrGUrArAUrGrGrGUTT; antisense, 5′rArCrCrCrAUUrArCrGrArArCUUrArArATT. For Epac1 knockdown, the siRNA (siEpac1) was designed with the following sequence: sense, 5′rAUrGrGrArCrCUrGUUUrGrGUUUrCrCUrATT; antisense, 5′UrArGrGrArArArCrCrArArArCrArGrGUrCrCrAUTT. The scrambled control siRNA for Epac1 (scEpac1) had the following sequence: sense, 5′UrCrGUUUrGrCrGrArCUUrAUrArCrGUUrGTT; antisense, 5′rCrArArCrGUrAUrArArGUrCrGrCrArArArCrGrATT. The siRNA for Epac2 knockdown (siEpac2) had the following sequence: sense, 5′rAUrCrCrGUrGrArAUrGUrArGUrCrAUUUrATT; antisense, 5′UrArArAUrGrArCUrArCrAUUrCrArCrGrGrAUTT. The scrambled control siRNA for Epac2 (scEpac2) had the following sequence: sense, 5′UUrAUrCrGUrCUrArAUrCrGrArGrAUrArGUTT; antisense, 5′rArCUrAUrCUrCrGrAUUrArGrArCrGrAUrArATT.

### Reagents

Sp-adenosine 3′,5′-cyclic monophosphorothioate triethylammonium salt hydrate (Sp-cAMPS) and Rp-adenosine 3′,5′-cyclic monophosphorothioate triethylammonium salt hydrate (Rp-cAMPS) were applied at 100 μM. ESI-09 (membrane-permeant inhibitor of Epac 1 and Epac 2, BIOLog) was applied at 10, 30 or 100 μM. Protein Kinase A inhibitor fragment 14–22 (PKI) was applied at 20 μM. N6-phenyladenosine- 3′,5′- cyclic monophosphate (6-phe-cAMP) was applied at 100 μM. 8-(4-chlorophenylthio)-2′-O-methyladenosine 3′,5′-cyclic monophosphate monosodium (8-cpt-2Me-cAMP) was applied at 200 μM.

### Blue-light stimulation

For all cultured cells, blue-light stimulation was applied using 20000-mcd LED lamps. Four LED lamps were connected in a parallel circuit with a 3-V DC power source. The intensity of the light stimulus was approximately 200 μmol/m^2^/s. The duration of the light pulse was set to 2 s, and the interval was set to 3 s. Each LED lamp was fixed to four corners of the lid of a 24-well plate 3 mm above the surface of the culture medium. For focal stimulation, illumination was performed using a FV1200 1 × 33 confocal laser scanning microscope (Olympus). Blue light at 472 nm was applied at 100% laser power for 1 min. The stimulation site was a circular region (Φ = 20 μm) on the axon. Each light stimulation was counted as 1 trial. Morphological changes were examined 1 h after stimulation. For each cell, 1 or 2 stimulation sites were randomly chosen. For each stimulation site, 5 consecutive trials were performed. Focal stimulation was performed from DIVs 4–6.

### Immunocytochemistry

Cultured neurons were fixed with 4% paraformaldehyde for 30 min at 4 °C. Fixed samples were washed 3 times with phosphate-buffered saline (PBS). The samples were then incubated with blocking solution (5% goat serum and 0.1% Triton-X 100 in PBS) for 1 h at room temperature. The samples were subsequently incubated with primary antibodies overnight at 4 °C. The samples were washed with PBS and incubated with secondary antibodies with or without the presence of rhodamine-conjugated phalloidin (1:50) for 6 h at room temperature. The primary antibodies were as follows: mouse anti-cAMP (1:1000, Abcam); mouse anti-mCherry (1:1000; Abcam); mouse anti-tau-1 (1:1000; Chemicon); rabbit polyclonal anti-EPAC1 antibody (1:1000; Abcam); rabbit polyclonal anti-EPAC2 antibody (1:1000; Abcam) rabbit anti-PAC (1:2000; from Dr. Iseki); rabbit anti-PKA alpha + beta polyclonal antibody (1:1000; Bioss); rabbit anti-Prox1 (1:1000; Chemicon); chicken anti-GFP (1:1000; Abcam); chicken anti-tau-1 (1:1000; Abcam); rabbit anti-PKA Reg I α/β (1:500; Santa Cruz); and rabbit anti-PKA Reg II (1:500; Santa Cruz).

The secondary antibodies were as follows: Alexa 488-labeled anti-mouse IgG (1:500; Invitrogen); Alexa 594-labeled anti-mouse IgG (1:500; Invitrogen); Alexa 405-labeled anti-rabbit IgG (1:500; Invitrogen); Alexa 594-labeled anti-rabbit IgG (1:500; Invitrogen); and Alexa 488-labeled anti-chicken IgY (1:500; Abcam).

### ELISA

The intracellular cAMP levels in HEK cells cultured on 24-well plates containing DMEM with 10% FBS were assayed using an enzyme immunoassay kit according to the manufacturer’s instructions (GE Healthcare).

### Time-lapse imaging

Images of fixed cells were acquired using a CellVoyager CV1000 confocal system (Yokogawa) with a 40× objective. Time-lapse imaging was performed using a FV1200 1×33 confocal scanning microscope (Olympus) and accompanying incubation system. Cells were incubated with 5% CO_2_ at 37 °C. For morphology analysis, tdTomato-fluorescence and differential interference contrast (DIC) images were acquired using a 559 nm excitation laser, which does not activate PAC^8^. For Fig. 2f, images were captured 1 h after light stimulation with a 40× objective. For Supplementary Fig. 5, images were captured 10 min after light stimulation for 30 min (5 min intervals) with a 40× objective.

### Morphology analysis and fluorescence quantification

Axon lengths were measured using ImageJ (NIH). The longest axon from each cultured neuron was defined as the primary axon, and the axon segments emerging from other axon segments were defined as axonal branches. Axonal branches shorter than 5 μm were excluded from the analysis. For new branching formation analysis, new branch density was defined as the newly formed branch number over the total length of the axon segments inside a specific region (at the stimulation site, as well as 0–10, 10–20, 20–30, and 30–40 μm from the stimulation site). Before stimulation, axons were measured to calculate the density. cAMP fluorescence intensity was calculated as the mean fluorescence intensity in the soma – the mean background fluorescence intensity. PKA, Epac1 and Epac2 intensities were measured at the primary axons, all collateral branches and the growth cones. The fluorescence ratio in the branches was defined as (mean fluorescence in branches – mean background fluorescence intensity)/(mean fluorescence in the primary axon – mean background fluorescence intensity). The fluorescence ratio in the growth cones was defined as (mean fluorescence in the growth cone – mean background fluorescence intensity)/(mean fluorescence in the primary axon – mean background fluorescence intensity). The fluorescence intensity of the growth cone was defined as the mean fluorescence intensity in a circular region (3 μm) at the primary axon tip. For dendritic morphology analysis, dendrites were reconstructed from cell images with tau, F-actin and prox1 labeling. Sholl analysis was performed using the FIJI software. The radii of concentric circles ranged from 10 μm to 115 μm, and the step size was 5 μm.

## Additional Information

**How to cite this article**: Zhou, Z. *et al*. Photoactivated adenylyl cyclase (PAC) reveals novel mechanisms underlying cAMP-dependent axonal morphogenesis. *Sci. Rep.* **6**, 19679; doi: 10.1038/srep19679 (2016).

## Supplementary Material

Supplementary Information
