# Supplementary material for: Photoactivated adenylyl cyclase (PAC) reveals novel mechanisms underlying cAMP-dependent axonal morphogenesis
Source: Sci Rep. 2016 Jan 22;5:19679. doi: 10.1038/srep19679 (PMC4726437; doi:10.1038/srep19679)
Supplement: Supplementary Information [file srep19679-s1.pdf]

Photoactivated adenylyl cyclase (PAC) reveals novel mechanisms underlying cAMP-dependent axonal morphogenesis

Zhiwen Zhou[1], Kenji F. Tanaka[2], Shigeru Matsunaga[3], Mineo Iseki[4], Masakatsu Watanabe[5], Norio Matsuki[1], Yuji Ikegaya[1], Ryuta Koyama[1*]

1. Laboratory of Chemical Pharmacology, Graduate School of Pharmaceutical Sciences, The University of Tokyo, 7-3-1 Hongo, Bunkyo-ku, Tokyo, Japan
2. Department of Neuropsychiatry, School of Medicine, Keio University, 35 Shinanomachi, Shinjuku, Tokyo, Japan
3. Central Research Laboratory, Hamamatsu Photonics K.K. , 5000 Hirakuchi Hamakita-ku, Hamamatsu, Shizuoka, Japan
4. Faculty of Pharmaceutical Sciences, Toho University, 2-2-1 Miyama, Funabashi, Chiba, Japan
5. The Graduate School for the Creation of New Photonics Industries, 1955-1 Kurematsu-cho, Nishiku, Hamamatsu, Shizuoka, Japan

* Correspondence should be addressed to Ryuta Koyama.
Laboratory of Chemical Pharmacology, Graduate School of Pharmaceutical Sciences, The University of Tokyo, 7-3-1 Hongo, Bunkyo-ku, Tokyo 113-0033, Japan
E-mail: rkoyama@mol.f.u-tokyo.ac.jp
Phone: +81-3-5841-4782
Fax: +81-3-5841-4786

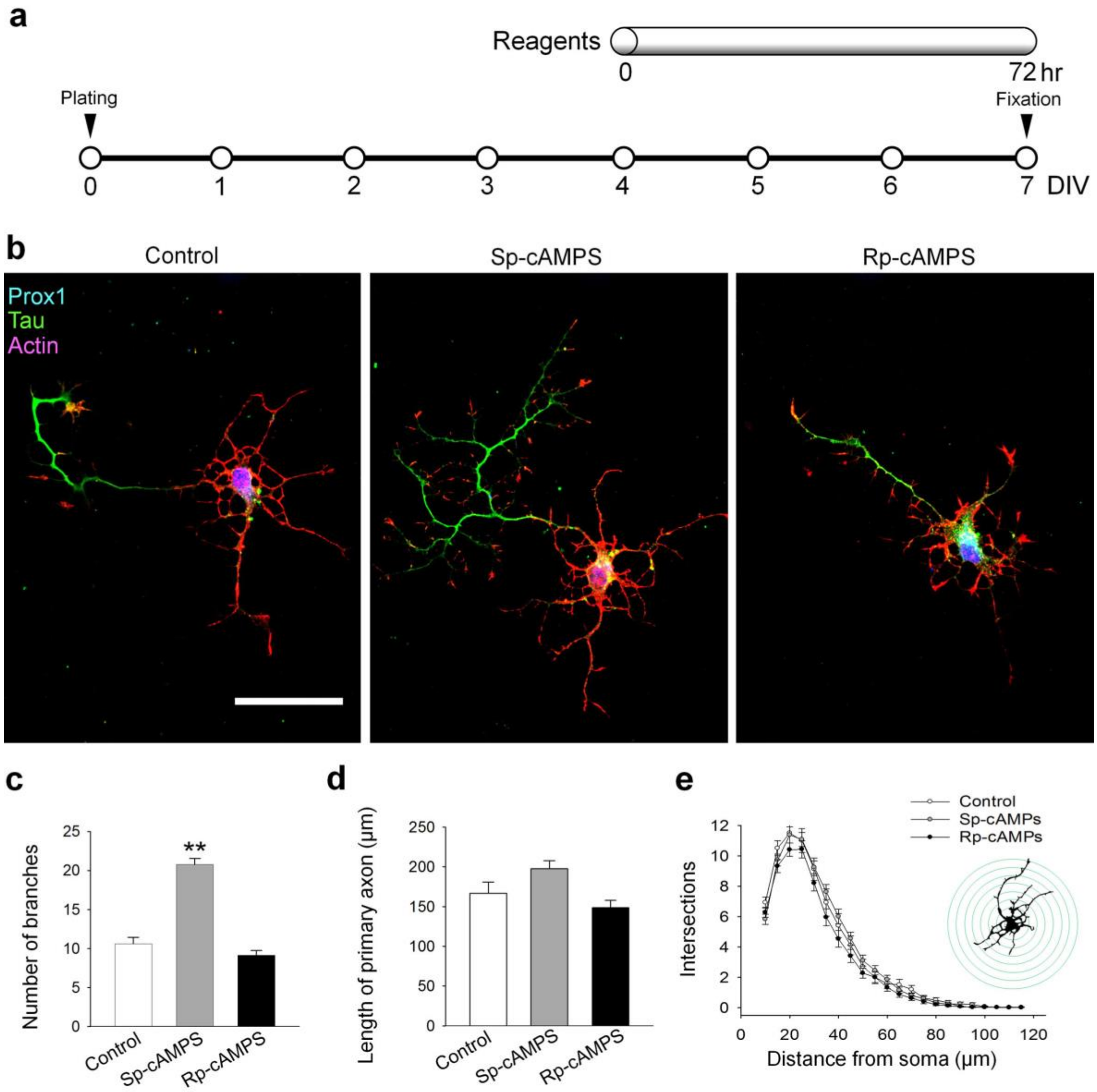

Supplementary Figure 1 | cAMP affected axonal but not dendritic morphology. (**a**) Experimental paradigms for the axonal morphology assay. Dissociated dentate granule cells were cultured for 7 days. The cAMP agonist Sp-cAMPS (100 μM) or the cAMP antagonist Rp-cAMPS (100 μM) was bath-applied from DIVs 4 to 7. Cells were fixed on DIV 7 for immunocytochemistry. (**b**) Representative images of granule cells cultured with or without reagents and immunostained for Prox1 (blue) and tau (green). Actin was labeled with rhodamine phalloidin (red). Scale bar = 50 μm. (**c**) Bar graphs indicating the number of branches. Sp-cAMPS, but not Rp-cAMPS, significantly increased the number of axonal branches. ***p* < 0.01 vs. control; Steel-Dwass test after Kruskal-Wallis test, n = 20-40 cells for each group. (**d**) Bar graphs indicating the length of the primary axon. Neither Sp-cAMPS nor Rp-cAMPS affected the length of the primary axon. Tukey's test after a one-way ANOVA, n =

20-40 cells for each group. (**e**) Dendritic morphology of cultured granule cells at DIV 7 was evaluated via Sholl analysis. The insert is an example of a reconstructed cultured cell with its dendrites intersected by several concentric circles. Outer circles were larger than the adjacent inner circles by 10 μm in radius. Plots of intersections revealed no significant difference between the control and the reagent-treated groups. Tukey's test after a one-way ANOVA. n = 40 cells for each group.

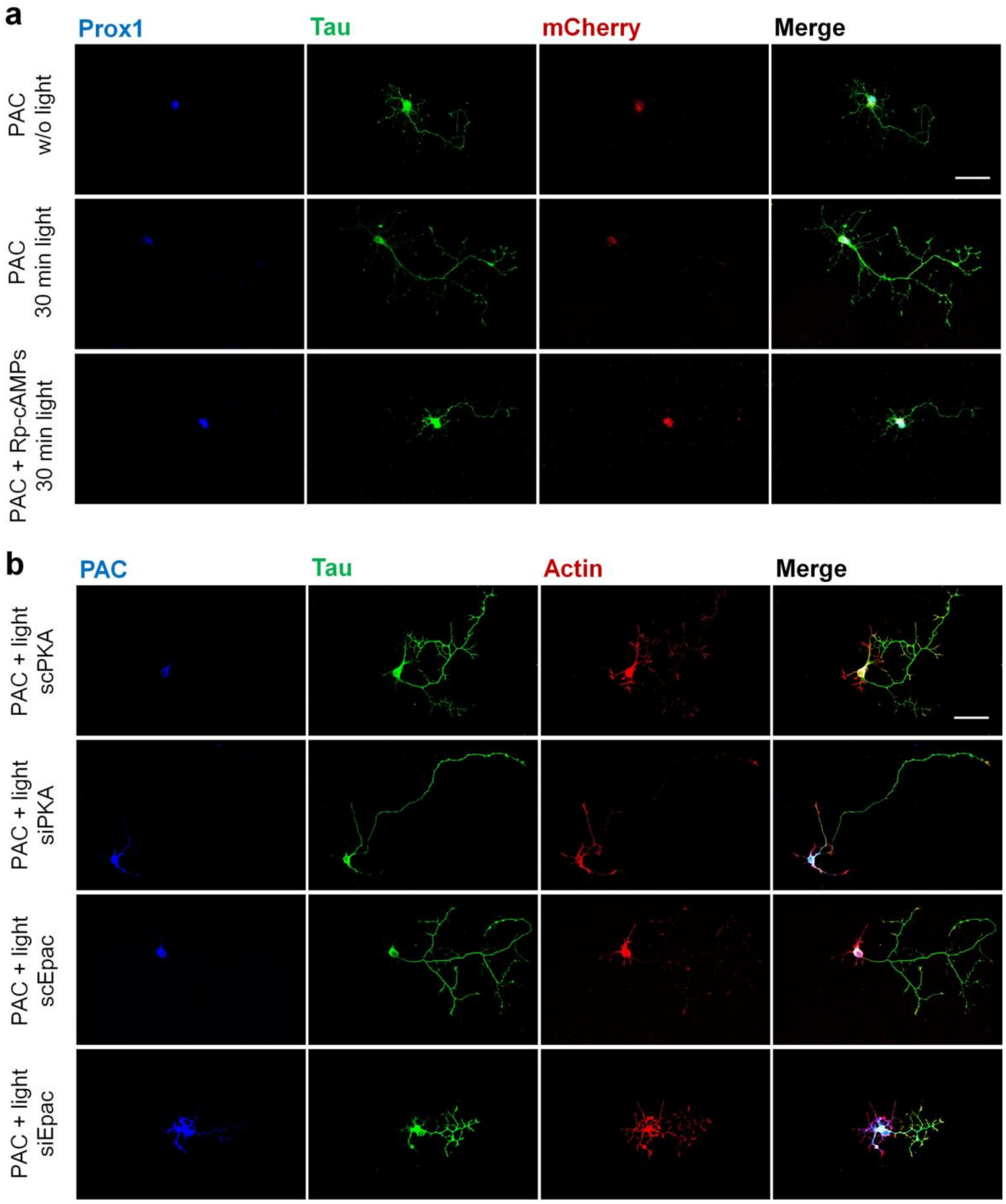

Supplementary Figure 2 | Representative immunocytochemical images of cultured granule cells in Figure 2 and Figure 4. (**a**) Representative granule cell images shown in Fig. 2b were shown in split channels: Prox1 (blue), Tau (green), and mCherry (red). Scale bar = 50 μm. (**b**) Representative granule cell images shown in Fig. 4b were shown in split channels: Prox1 (blue), Tau (green), and Actin (red). Scale bar = 50 μm.

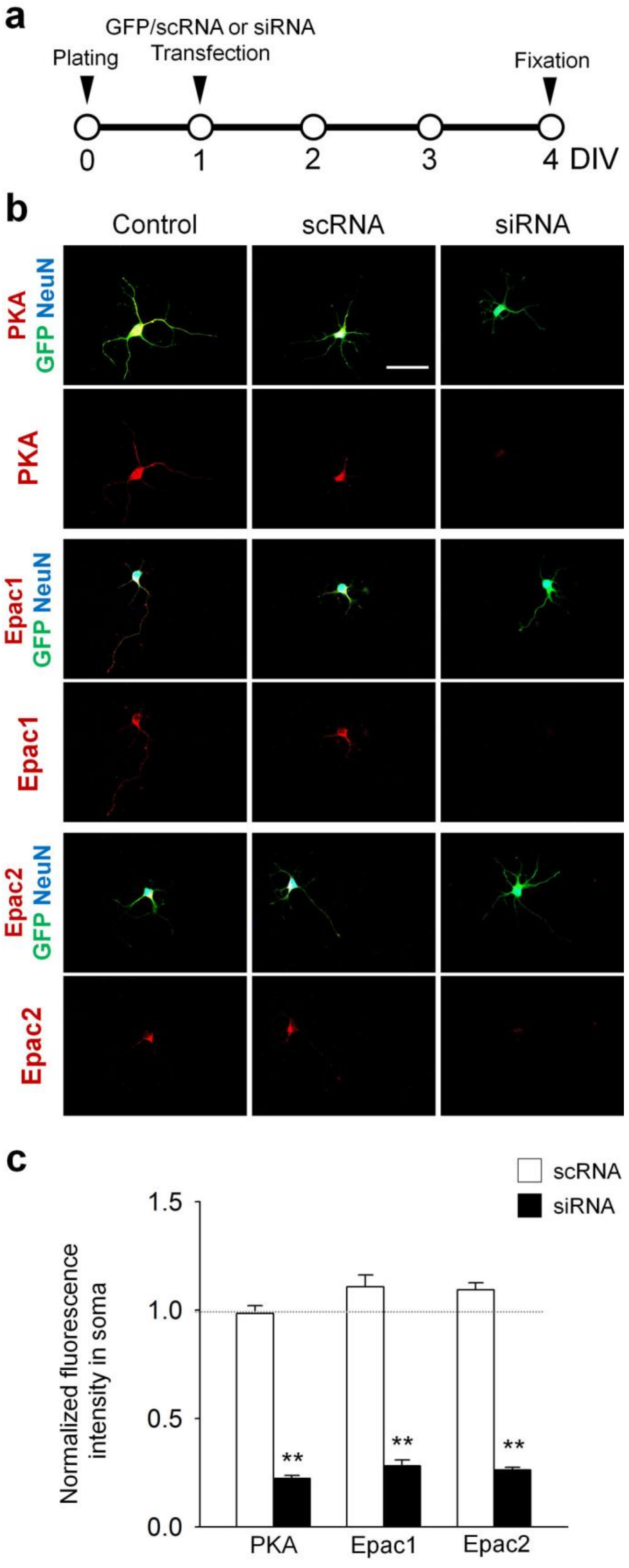

Supplementary Figure 3 | Evaluation of siRNAs for the knockdown of PKA and Epac. (**a**) Experimental paradigms for the immunocytochemical evaluation of the knockdown efficiencies of PKA, Epac1, and Epac2. Dissociated dentate granule cells were transfected with siRNAs against the PKA α and β subunits to knockdown PKA or against Epac1 and Epac2 to knockdown Epac on DIV 1. scRNAs for PKAα, PKAβ, Epac1, and Epac2 were transfected for control experiments. DNA constructs encoding GFP were co-transfected with the scRNAs or siRNAs to visualize the transfected cells. Cells were fixed for immunocytochemistry on DIV 4. (**b**) Representative images of cultured dentate granule cells immunostained for the neuronal marker NeuN (blue), GFP (green), and PKA, Epac1, or Epac2 (red). Scale bar = 50 μm. (**c**) The quantification of fluorescence intensity in the soma reveals that PKA, Epac1 and Epac2 were efficiently knocked down by siRNAs, whereas the control scRNAs did not affect the expression level of each protein. The fluorescence intensities of scRNA- or siRNA-transfected cells were normalized to those of the control cells. ** $p < 0.01$ vs. scRNA; Tukey's test after a one-way ANOVA, n = 30 for each group.

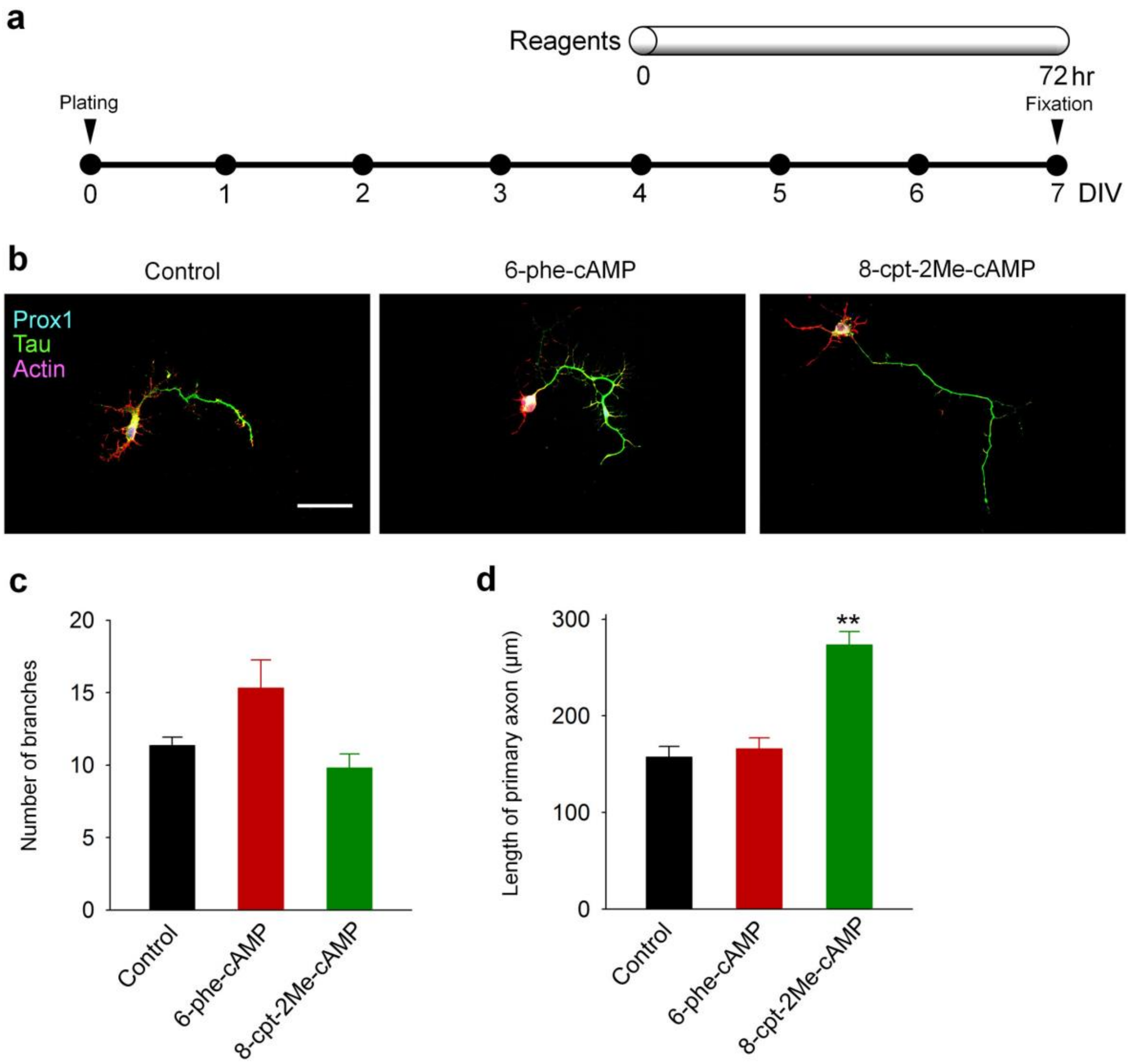

Supplementary Figure 4 | Pharmacological activation of PKA and Epac promotes axonal morphogenesis. (**a**) Experimental paradigms for the axonal morphology assay. Dissociated dentate granule cells were cultured for 7 days. The PKA activator 6-Phe-cAMP (200 μM) or the Epac activator 8-cpt-2Me-cAMP (200 μM) was bath-applied from DIVs 4 to 7. Cells were fixed on DIV 7 for immunocytochemistry. (**b**) Representative images of granule cells cultured with or without reagents and immunostained for Prox1 (red) and tau (green). Actin was labeled with rhodamine phalloidin (red). Scale bar = 50 μm. (**c**) Bar graphs indicating the number of branches. **p < 0.01 vs. control; Steel-Dwass test after Kruskal-Wallis test, n =20- 30 cells for each group. (**d**) Bar graphs indicating the length of the primary axon. **p < 0.01 vs. control; Tukey's test after a one-way ANOVA, n = 20-30 cells for each group.

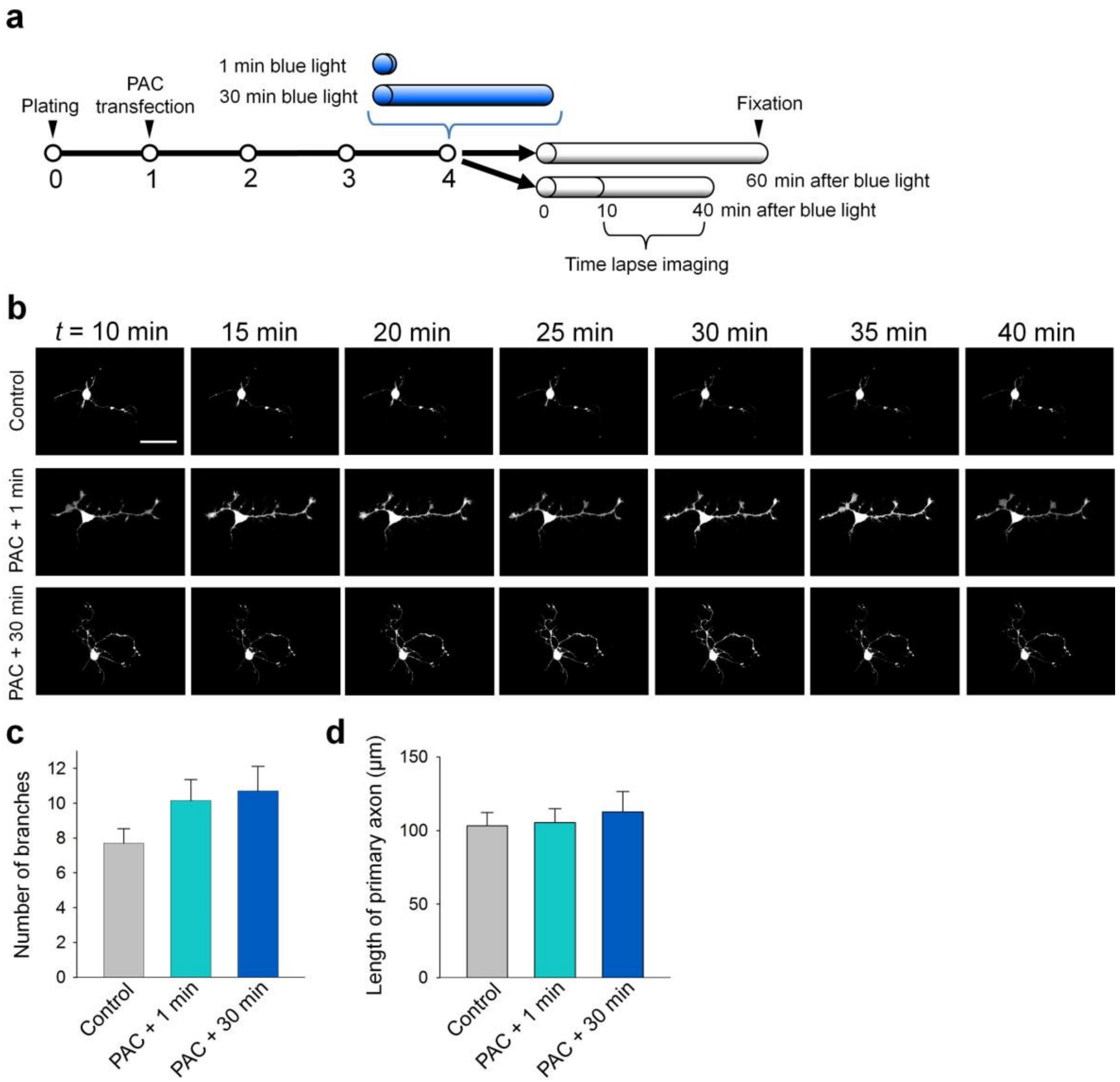

Supplementary Figure 5 | Neither 1 min nor 30 min cAMP elevation result in immediate axonal elongation. (**a**) Experimental paradigms for the axonal morphology assay. Cultured cells were exposed to 1 min or 30 min blue light at DIV 4. Then some cells were used for 30 min time-lapse imaging 10 min after the blue light stimulation. The other cells were fixed 1h after the blue light stimulation for immunocytochemistry. The cells used for time-lapse imaging were transfected with membrane targeted-tdTomato for visualization. (**b**) Representative time-lapse images of cultured granule cells 10 min after the blue light stimulation. Cell morphology was visualized by tdTomato fluorescence, and the images were taken every 5 min. Scale bar = 50 μm. (**c**) Bar graphs indicating the number of branches. No significant difference was detected between Control, PAC + 1 min, and PAC + 30 min groups. Kruskal-Wallis test, n = 20 cells for each group. (d) Bar graphs indicating the length of the primary axon. No significant difference was detected between Control, PAC + 1 min, and PAC + 30 min groups. One-way ANOVA, n = 20 cells for each group.
